# Detroit under siege, the enemy within: The impact of the COVID-19 collision

**DOI:** 10.1017/ice.2020.154

**Published:** 2020-04-21

**Authors:** Teena Chopra, Jack Sobel

**Affiliations:** Wayne State University, Detroit, Michigan

*To the Editor*—Our Detroit is burning. It is not a blaze that is scorching the city we love, but an invisible enemy that has the city under siege. As COVID-19 spreads its tentacles deeper into the lives of our people, we scramble to fight this new inferno in a community that is still recovering from an unfortunate past. One of the shrinking cities of the United States, Detroit has faced urban decay due to a multitude of socioeconomic factors. A dramatic decline in population, loss of industrial and working-class jobs, and vanishing businesses have all hit our city hard in the last several decades.

Although younger people moved out of the city, most of the 700,000 stayed behind. The remaining population is disproportionately elderly lacks resources and transportation, and is heavily dependent on state help.^1,^ ^2^ Today, 36% of Detroiters live in poverty—1 in 3 people in the city that is home to Motown and the world’s original automotive manufacturing center. With the city’s bankruptcy in 2018 came the disintegration of a failing healthcare system, and in fiscal recovery, the critical public health framework and its safety net have not followed.

With a median income of $26,000, far below the US average, we have struggled with socioeconomic and healthcare disparities. Nowhere are the social determinants of health as glaring and impactful as in Detroit. The high rates of unemployment and medically uninsured, in addition to reliance on a dramatically reduced auto industry for jobs, all underpin the current invasion by the COVID-19 pandemic. The lockdown poses a huge barrier for daily wage earners; it is an enormous complicating factor in this dire situation.

In the past decade, private investors and companies have begun to resurrect Detroit by pumping new life and confidence into the economy. As their efforts began to catalyze progress, little did they know that a lethal enemy would strike down this regrowth like a tsunami.

As COVID-19 ravages our city, our people are hit hard. A population with an overburdened healthcare system that is underserved socially, fiscally, and educationally is ill equipped to tackle this deadly adversary. With cases doubling daily and mortality dramatically escalating, we have become the next epicenter in the United States. The growth of the infection rate and the total cumulative numbers of cases in Detroit are high, with a curve steeper than that of New York due to the dangerous combination of inadequate resources and higher rates of comorbid conditions like hypertension, diabetes, and obesity. To top it off, testing has been massively inadequate. Our vulnerability puts us in a very unfortunate situation. SARS-CoV-2 has revealed the previously unrecognized susceptibility of a vulnerable community unaware of its precarious situation.

With the aggressive efforts of medical personnel and our amazing nurses and hospital staff, we will continue to combat this steep curve. COVID-19 will, regrettably, take a massive toll in Detroit. Poverty, inadequate healthcare, higher rate of macro and microvascular disease, and an inherent mistrust of the medical community make our beloved city a perfect storm for this pandemic. At highest risk are the elderly, especially those in nursing homes.

We continue to rely on appeals for social distancing and watching out for the elderly, the herculean efforts of our healthcare providers, and the generosity of philanthropists. Detroit is a community built on care. We count on the spirit of our people—their resilience, courage, and ability to rise up in the face of adversity. We start each day with a renewed vigor to fight the microbial opponent and aggressive efforts to safeguard the health of our people. In the face of this perfect storm, we pray and hope that no matter what, we will ultimately contain this contagion. Can we count on the grit of this great city to bounce back yet again when the world reboots after this pandemic passes?
